# Parliament and the Professions

**Published:** 1919-06-14

**Authors:** 


### PARLIAMENT AND THE PROFESSIONS.

### Venereal Disease.

Mr. Winston Churchill, in reply to a question by Sir Thomas Bramsdon, said that a report had been called for in respect of the alleged spread of venereal disease amongst the British troops in Germany and the greater incidence of disease amongst troops returning to England.

### Hospital Sites in London.

Commander Bellairs asked whether the sites of London hospitals have ever been considered as a whole; whether the Ministry of Health will have authority to produce co-ordination, so as to secure that hospitals shall be gradually moved to the sites best suited to modern conditions; and whether the decision of the St. George's Hospital Committee to extend the present hospital will receive early consideration in view of the desirability of shifting the site of the hospital. Dr. Addison stated that he was not aware that the matter referred to in the first paragraph of the question has yet been taken in hand, and that the decision of St. George's Hospital is not one on which the Local Government Board has any power to intervene.

### Certified Medical Supplies of Spirits.

Sir Fortescue Flannery asked the Food Controller whether his attention has been called to the difficulty, still continuing, of obtaining certified medical supplies of brandy and whisky for invalids. Mr. Roberts stated that, according to the information at his disposal, the quantities of spirits now permitted to be released from bond should be sufficient for the reasonable requirements of the public. The question of modifying the present restrictions is one for the War Cabinet.

